# Blood pressure reverse dipping may associate with stable coronary artery disease in patients with essential hypertension: a cross-sectional study

**DOI:** 10.1038/srep25410

**Published:** 2016-05-03

**Authors:** Bin Yan, Lu Sun, Ya Gao, Qi Guo, Litao Guo, Xue Wang, Gang Wang

**Affiliations:** 1 Department of Emergency Medicine, the Second Affiliated Hospital, Xi’an Jiaotong University, Xi’an, China; 2 Department of Ultrasound, the Second Affiliated Hospital, Xi’an Jiaotong University, Xi’an, China; 3 Department of Cardiology, the Second Affiliated Hospital, Xi’an Jiaotong University, Xi’an, China; 4 Intensive Care Unit, the First Affiliated Hospital, Xi’an Jiaotong University, Xi’an, China

## Abstract

The dipping variations of circadian blood pressure (BP) correlate closely with target-organ damages and cardiovascular events. The aim of this study was to investigate the relationship between BP reverse dipping and the prevalence of stable coronary artery disease (sCAD) in hypertensive patients. Clinical data and the results of 24-hour ambulatory BP monitoring (ABPM) were obtained from 718 hypertensive patients (390 males, mean age 59.6 ± 13.8 years) in a single centre in Northern China. Reverse dipping pattern was defined as nocturnal systolic BP (SBP) was higher than daytime SBP. A logistic regression model was applied to explore the independent risk factors of sCAD. The patients with BP reverse dipping accounted for 31.5% in sCAD group and 19.5% in control group (*P* < 0.05). In multivariate analysis, BP reverse dipping remained significantly associated with the prevalence of sCAD (Odds ratio [OR], 1.772; *p* = 0.027). Furthermore, the circadian decline rate of SBP was independently associated with sCAD (OR, 0.975; *p* = 0.043). The hypertensive patients with reverse BP dipping were found to be more frequently suffering from sCAD. BP reverse dipping examined with 24-hour ABPM may indicate sCAD.

Blood pressure (BP) presents a reproducible circadian pattern, which is normally lower at night than during the day by approximately 10–20%, mostly due to endogenous neuroendocrine rhythms and other variables^1^. Circadian BP variations have been gradually realized as an important predictor for a range of organ damages and cardiovascular events^2^,^3^,^4^. According to the nocturnal BP dipping, circadian BP patterns are classified as extreme dipping (night/day ratio ≤ 0.8), dipping (0.8< ratio ≤ 0.9), nondipping (0.9< ratio ≤ 1.0) and reverse dipping (ratio >1.0)^5^. BP reverse dipping, with higher mean night SBP than daytime, was previously categorized as part of “nondipping”^6^,^7^. Recently, the growing numbers of studies have shown that BP reverse dipping is an independent risk factor for a range of cardiovascular and other morbidities^7^,^8^,^9^.

Our previous studies have demonstrated that BP reverse dipping was exposed to higher risks for lacuna infarction and carotid plaque formation, which was shown to be able to predict coronary artery disease (CAD) accurately^10^,^11^,^12^. CAD is the most common type of cardiovascular disease, resulting in millions of death across the world^13^. It is well substantiated that hypertension is one of the most important risk factors for CAD^14^. Furthermore, while blunted nocturnal BP dipping has also been proved to be a cardiovascular risk predictor, a previous study reported myocardial ischemia might follow certain type of circadian BP changes^6^,^15^.

There are published evidences indicating carotid plaque could predict ischemic stroke and CAD accurately in clinical practice^11^,^16^,^17^. Therefore, we hypothesize a potential link between reverse dipping of BP and CAD. The aim of this cross-sectional study was to investigate whether or not the hypertensive patient with BP reverse dipping status had higher prevalence of stable CAD (sCAD). The results may further strengthen the necessity of circadian BP rhythm administration, especially in hypertensive patients with reverse dipping.

## Results

The age of all hypertensive patients (257 sCAD and 461 non-sCAD) is 59.6 ± 13.8 years (range: 21 to 87) in average, 54.3% of the participates were males, 29.9% were current smokers, 26.3% had diabetes mellitus and 24-hour SBP were 134.6 ± 14.0 mmHg. Hypertensive patients with sCAD (31.5%) had higher percentage of BP reverse dipping pattern than control group (19.5%) (p = 0.004). In the meanwhile, the proportion of dipping pattern was 18.3% in sCAD group and 28.2% in non-sCAD group. Moreover, the ratio of nondipping pattern was similar between sCAD (50.2%) and non-sCAD (52.3%). Other clinical characteristics of the participates are shown in Table 1. There was no gender difference in the circadian BP patterns (Supplement Table 1).

**Figure 1:**
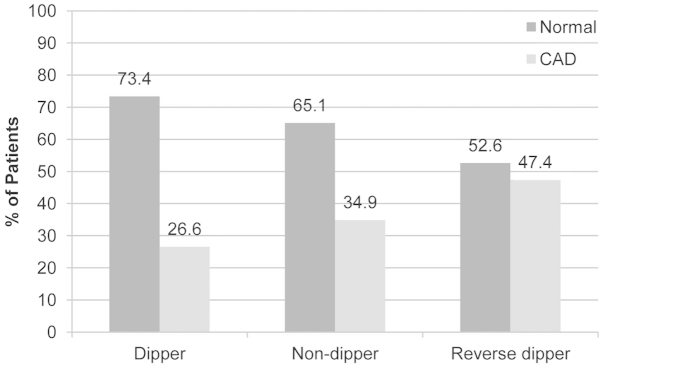
The distribution of sCAD in each circadian BP pattern group. The difference between dipping and nondipping, dipping and reverse dipping, nondipping and reverse dipping were statistically significant (*P* = 0.031, *P* < 0.001 and *P* = 0.004), respectively.

The distribution of hypertensive patients with sCAD in dipping, nondipping and reverse dipping group was 26.6%, 34.9% and 47.4%, respectively. After using chi-squared test, the difference between dipping and non-dipping (*p* = 0.031), dipping and reverse dipping (*p* < 0.001), non-dipping and reverse dipping (*p* = 0.004) were statistically significant. Patients with reverse dipping pattern had a lowest prevalence of non-sCAD while had a highest prevalence of sCAD (Fig. 1, *p* < 0.01).

**Figure 2:**
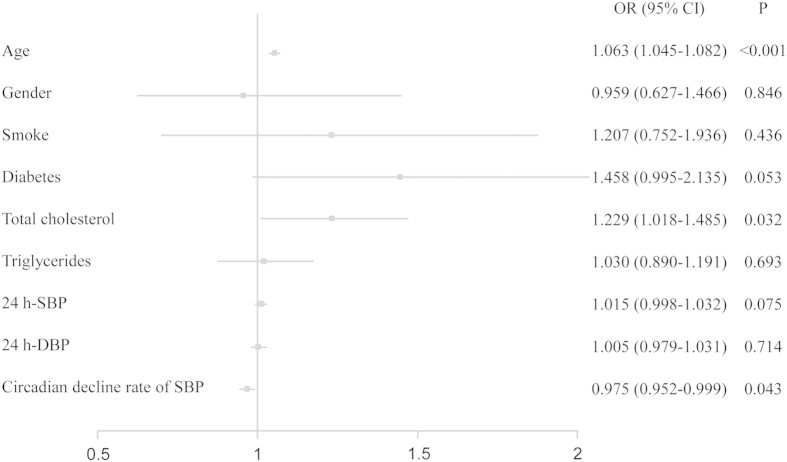
Multivariate logistic regression analysis for the presence of stable coronary artery disease.

In univariate analysis, the risk factor related to the presence of sCAD were age, gender, smoking, diabetes, cholesterol, triglycerides, 24-hour SBP, 24-hour DBP, nondipping and reverse dipping of BP. For further multivariate logistic regression analysis, BP reverse dipping (OR = 1.772, *P* = 0.027), age (OR = 1.064, *P* < 0.001) and total cholesterol (OR = 1.226, *P* = 0.035) were significantly correlated with sCAD (Table 2). In addition to this, circadian decline rate of SBP (OR = 0.975, *P* = 0.043) was significantly related to sCAD (Fig. 2).

Our previous study revealed that diabetes and reverse dipping were interrelated risk factor for each other. This study also found that diabetes (OR = 1.588, *P* = 0.020) and age (OR = 1.034, *P* < 0.001) were significantly associated with reverse dipping (Supplement Table 2). After exclusion of sCAD patient with diabetes, reverse dipping was still an independent risk factor for sCAD (OR 1.673, *P* = 0.038; data not shown).

## Discussion

Hypertension is a well-recognized risk factor for CAD^14^. Although not written into guidelines, amount of evidences indicated the fluctuations of BP over a certain period could provide additional prognostic value. For example, visit-to-visit BP variability, a type of long-term variability, was previously considered to be non-specific but now shown to be associated with stroke, in most studies, if not all^18^. Circadian and minute-to-minute BP variability revealed by ABPM have both been studied and gradually recognized as important cardiovascular risk factors as well^2^,^19^. ABPM is a noninvasive examination of circadian BP over a span of 24 hours, providing valuable diagnostic information for patients with fluctuating BP and circadian BP profile^20^,^21^,^22^.

Circadian BP variations were used to be divided into dipper (mean nocturnal BP drops 10 mmHg or more than that in daytime) and “non-dipper”^23^,^24^. Previously, there has been a series reports demonstrating the relationship between “non-dipper” and cardiovascular complications such as lacunar infarction, metabolic syndrome and myocardial ischemia in hypertensive patients with CAD^15^,^24^,^25^. Other reports also stressed on the important prognostic value of “non-dipper”^25^,^26^,^27^. Unfortunately, conflicts exist, especially when comparing our clinical observations with those findings.

We have previously found “non-dippers” had significant heterogeneity in complications among a small population of hypertensive patients. To further clarify the impact of different circadian BP patterns, we divided the patients into normal dipping (night-to-day SBP ratio ≤ 0.9 and >0.8), extreme dipping (night-to-day SBP ratio ≤ 0.8), nondipping (night-to-day SBP ratio ≤ 1.0 and >0.9) and reverse dipping (night-to-day SBP ratio >1.0) according to previous literature^28^. After analysis of multivariate logistic regression, reverse-dipper pattern (OR 2.677; 95%CI 1.226–5.842; *p* < 0.05), not non-dipper pattern, was directly associated with lacunar infarction^12^.

As a particular portion of non-dipper pattern, reverse dipper pattern has been shown to correlate with the highest incidence of cardiovascular events and worst prognosis in hypertensive patients, comparing with the dippers and non-dippers^5^. Our cross-sectional studies in patients comprising dipper, non-dipper and reverse dipper groups further confirmed, instead of non-dipper, nocturnal rise of BP may associate with MetS in male^20^ and early formation of carotid plaque in senior hypertensive patients^10^. Additionally, in the current study, our results showed that reverse dipper pattern of BP was positively associated with the prevalence of sCAD. Therefore, reverse dipper pattern of BP may be an independent risk factor to predict the incidence of CAD.

Although the relationship may be present between reverse dipper pattern of BP and CAD, the pathophysiological mechanism is far more to be investigated. We would like to propose that the higher risk of atherosclerosis associated with reverse dipper pattern of BP may relate to relatively higher blood mechanical forces on endothelial cells, leading to the damage of vascular integrity. It has been revealed that BP variability may correlate with aortic wall remodeling and reduced aortic compliance in the animal experiment^29^. On the other hand, the sensitivity of arterial baroreceptor could be compromised due to large arterial stiffness and resulted in abnormal BP variability^18^. Another report suggested that coronary artery calcium might serve as a potential mediator involved in the relationship between abnormal nighttime BP pattern and cardiovascular disease^30^. In addition, the Sympathetic Nervous System (SNS) also plays important roles in the regulation of blood pressure and chronic SNS overactivity could contribute to the development of hypertension and may change the BP pattern^31^. SNS also result in an elevated inflammatory response and destabilization of atherosclerotic plaques^32^. Therefore, the SNS may be a significant link between BP variation and CAD, which worth to be studied in the future^33^,^34^.

Ultrasound assessment of carotid plaque is a non-invasive imaging test to evaluate the cardiovascular complications of patient^35^. Ellisiv *et al.*^16^ suggested that total plaque area and carotid intima-media thickness (CIMT), were closely related to ischemic stroke. Moreover, accumulating evidences indicated that the carotid plaque represent atherosclerotic pathogenesis more accurately than CIMT^17^. Previous meta-analysis also revealed that the ultrasound detection of carotid plaque had a higher prognostic accuracy for the prediction of future CAD events than CIMT^11^. Together with our previous discovery about reverse BP dipping and carotid plaque, it is reasonable to hypothesize that reverse dipping may serve as a risk factor for CAD. Therefore, future prospective studies in human are needed to look into the potential network of regulation^36^.

To our knowledge, this is the first study to report the association between reverse dipper pattern of BP and the incidence of sCAD in individuals with hypertension. Therefore, the development of antihypertensive medicine with an effect on decreased fall of nocturnal BP may influence the development of sCAD. Further studies are needed to address the differing impact of extreme-dipper pattern on CAD in hypertensive patients. In addition, multiple ABPM over a longer period of time may provide more information.

## Methods

### Study Population

**Figure 3:**
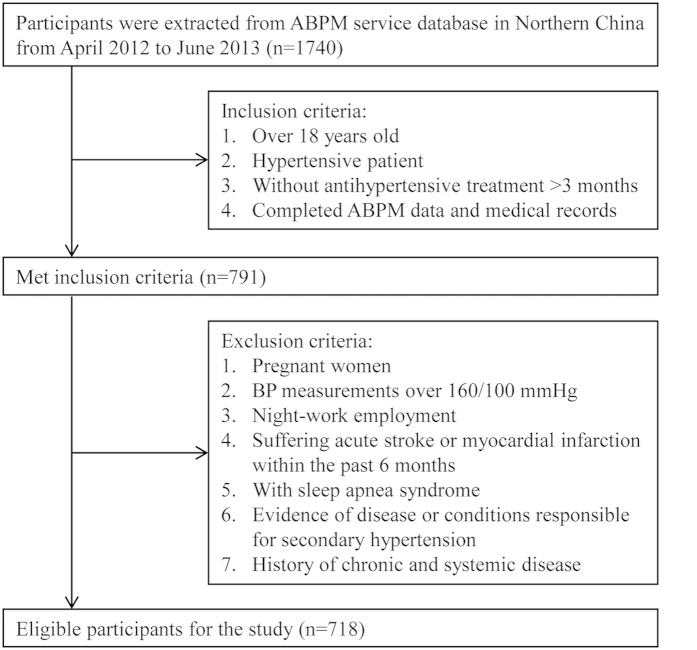
Flow diagram for the selection of patient.

Hypertensive patient selection and data retrieval were extracted from ABPM database from April 2012 to June 2013 (n = 1740) in the Second Affiliated Hospital, Xi’an Jiaotong University School of Medicine, Northern China. Hypertensive patients were defined as office BP ≥ 140/90 mmHg or 24-hour ABPM ≥ 135/85 mmHg^37^. Because the treatments of hypertension have influence on the circadian BP variation, we selected patients who were not treated with antihypertensive drug or first detected hypertension. 718 patients (390 men and 328 women) with essential hypertension who fulfilled exclusion criteria were eventually included in our cross-sectional study (Fig. 3). All patients were referred due to standard indications that have been shown to use ABPM for appropriate clinical circumstances^20^,^38^. ABPM were carried out for diagnosis of hypertension and assessment of vascular risk in adults with the approved guidelines^39^. The study protocol was approved by the Ethics Committee of the Second Affiliated Hospital, Xi’an Jiaotong University School of Medicine. All the participants read the purpose statement of the investigation and each provided a written informed consent.

### ABPM measurement

Ambulatory BP was monitored over a 24-hour period using an oscillometric device (Spacelabs 90207; Spacelabs, Redmond, WA, USA). All the participants were asked to record their activities, sleep times and sleep quality during the monitoring session. Strenuous physical activity was discouraged during the monitoring period. The BP recording was made every 30 min from 7:00 AM till 11:00 PM and every 60 min from 11:00 PM till 7:00 AM. The parameters evaluated were mean 24-hour systolic and diastolic BP (SBP and DBP), SBP- and DBP-awakening, SBP- and DBP-bedtime. We divided the patient according to nocturnal BP reduction as follow: normal dipping (night-to-day SBP ratio ≤ 0.9 and >0.8), extreme dipping (night-to-day SBP ratio ≤ 0.8), nondipping (night-to-day SBP ratio ≤ 1.0 and >0.9) and reverse dipping (night-to-day SBP ratio >1.0)^28^.

### The diagnosis and assessment of sCAD

The initial diagnostic approach for CAD encompasses syndrome, identifying significant dyslipidaemia, hyperglycaemia or other biochemical risk factors, chest radiography, echocardiography and an electrocardiogram. Once this initial evaluation is performed stress testing, CT angiography and a coronary angiogram may be necessary to obtain further diagnostic insight^40^.

### Statistical Analysis

All the data was analyzed using SPSS version 18.0 (SPSS Inc., Chicago, IL, USA). Bivariate comparisons between patients with and without sCAD were performed by unpaired *t*-test (continuous variables) and by χ^2^-test (categorical variables), respectively. A logistic regression model was utilized to analyze the relationship between sCAD and age, gender, smoking, diabetes, cholesterol, triglycerides, circadian blood pressure variation and ABPM results. Variables with statistical significance in univariate models were then included in the multivariate analyses. A two-tailed P value of less than 0.05 was considered statistically significant.

## Additional Information

**How to cite this article**: Yan, B. *et al.* Blood pressure reverse dipping may associate with stable coronary artery disease in patients with essential hypertension: a cross-sectional study. *Sci. Rep.* **6**, 25410; doi: 10.1038/srep25410 (2016).

## Supplementary Material

Supplementary Information

## Figures and Tables

**Table 1:** Baseline characteristics of patients.

Variable	All (n = 718)	sCAD (n = 257)	Non-sCAD (n = 461)	P value
Clinical				
Age, years	59.6 ± 13.8	66.4 ± 11.0	55.8 ± 13.7	<0.001
Male/female	390/328	128/129	262/199	0.073
Smoking, n (%)	215(29.9)	91(35.4)	124(26.9)	0.011
Diabetes, n (%)	189(26.3)	82(31.9)	107(23.2)	0.013
Laboratory data				
Fasting glucose, mmol/L	5.4 ± 2.0	5.5 ± 2.5	5.3 ± 1.7	0.290
Triglycerides, mmol/L	1.8 ± 1.3	2.0 ± 1.6	1.7 ± 1.1	0.016
Total cholesterol, mmol/L	4.6 ± 1.0	4.7 ± 1.0	4.5 ± 1.0	0.001
HDL-C, mmol/L	1.2 ± 0.3	1.3 ± 0.3	1.2 ± 0.3	0.857
LDL-C, mmol/L	2.7 ± 0.9	2.8 ± 0.9	2.6 ± 0.9	0.098
VLD-C, mmol/L	0.7 ± 0.5	0.7 ± 0.6	0.6 ± 0.5	0.067
ABPM results				
24 h-SBP, mmHg	134.6 ± 14.0	136.5 ± 14.3	133.9 ± 13.8	0.018
SBP-awakening, mmHg	136.4 ± 13.9	138.1 ± 14.4	135.0 ± 13.7	0.005
SBP-bedtime, mmHg	130.0 ± 17.2	130.2 ± 16.4	129.7 ± 17.8	0.729
24 h-DBP, mmHg	79.4 ± 9.8	81.5 ± 10.7	76.3 ± 9.2	<0.001
DBP-awakening, mmHg	82.5 ± 10.8	82.9 ± 10.3	77.2 ± 9.4	<0.001
DBP-bedtime, mmHg	73.9 ± 10.3	76.3 ± 11.0	72.3 ± 10.0	<0.001
Circadian blood pressure				<0.001
Reverse-dipper, n (%)	171(23.8)	81(31.5)	90(19.5)	–
Non-dipper, n (%)	370(51.5)	129(50.2)	241(52.3)	–
Dipper, n (%)	177(24.7)	47(18.3)	130(28.2)	–

Results are presented as mean ± standard deviation or n (%). The P values represent the differences between sCAD and non-sCAD.

ABPM, ambulatory blood pressure monitoring; DBP, diastolic blood pressure; HDL-C, high-density lipoprotein cholesterol; LDL-C, low-density lipoprotein cholesterol; sCAD, stable coronary artery disease; SBP, systolic blood pressure; VLD-C, very low density lipoprotein cholesterol.

**Table 2:** Univariate and multivariate logistic regression analysis for stable coronary artery disease.

Variable	Univariate regression analysis		Multivariate regression analysis	
	OR (95% CI)	P value	OR (95% CI)	P value
Age	1.068 (1.054–1.083)	<0.001	1.064 (1.045–1.082)	<0.001
Gender	0.754 (0.555–1.024)	0.070	0.959 (0.627–1.466)	0.846
Smoke	1.562 (1.106–2.207)	0.011	1.207 (0.752–1.936)	0.436
Diabetes	1.550 (1.103–2.178)	0.011	1.454 (0.992–2.131)	0.055
Total cholesterol	1.897 (1.337–2.691)	<0.001	1.226 (1.014–1.483)	0.035
Triglycerides	1.185 (1.035–1.358)	0.014	1.027 (0.888–1.188)	0.720
24 h-SBP	1.013 (1.002–1.025)	0.019	1.015 (0.999–1.032)	0.074
24 h-DBP	1.054 (1.036–1.071)	<0.001	1.005 (0.979–1.032)	0.685
Reversed-dipper pattern	2.489 (1.589–3.899)	<0.001	1.772 (1.068–2.937)	0.027
Non-dipper pattern	1.481 (0.996–2.200)	0.052	1.365 (0.882–2.114)	0.163

95% CI, 95% confidence interval; DBP, diastolic blood pressure; OR, odds ratio; SBP, systolic blood pressure.
