# Supplementary material for: Blood pressure reverse dipping may associate with stable coronary artery disease in patients with essential hypertension: a cross-sectional study
Source: Sci Rep. 2016 May 3;6:25410. doi: 10.1038/srep25410 (PMC4853743; doi:10.1038/srep25410)
Supplement: Supplementary Information [file srep25410-s1.pdf]

Blood pressure reverse dipping may associate with stable coronary artery disease in patients with essential hypertension: a cross sectional study

Bin Yan[1], Lu Sun[2], Ya Gao[1], Qi Guo[3], Litao Guo[4], Xue Wang[4], Gang Wang[1]

Supplement Table 1 Characteristics of the study population by gender

Variable	Male	Female	P value
Patients, n	390	328	
Age, years	58.1±14.9	61.4±12.1	0.001
Current smokers, n (%)	211(54.1)	4(1.2)	0.001
Diabetes, n (%)	110(28.2)	79(24.1)	0.122
Triglycerides (mmol/L)	2.0±1.6	1.8±1.2	0.126
Total cholesterol (mmol/L)	4.5±1.0	4.8±1.0	0.003
HDL-C	1.2±0.4	1.3±0.3	0.001
LDL-C	2.7±0.9	2.8±0.8	0.232
VLD-C	0.7±0.6	0.7±0.6	0.776
24 h-SBP, ABPM (mmHg)	137.6±14.2	133.1±13.8	0.001
24 h-DBP, ABPM (mmHg)	82.4±10.0	76.3±10.1	0.001
Circadian BP pattern, n (%)			0.761
Dipper	100(25.6)	77(23.5)	—
Non-dipper	200(51.3)	170(51.8)	—
Reverse dipper	90(23.1)	81(24.7)	—

ABPM, ambulatory blood pressure monitoring; DBP, diastolic blood pressure; HDL-C, high-density lipoprotein cholesterol; LDL-C, low-density lipoprotein cholesterol; SBP, systolic blood pressure; VLD-C, very low density lipoprotein cholesterol.

Supplement Table 2 Univariate and multivariate logistic regression analysis for reverse dipping

	Univariate regression analysis		Multivariate regression analysis	
Variable	OR (95% CI)	P	OR (95% CI)	P
Age	1.041 (1.027-1.056)	<0.001	1.034 (1.018-1.050)	<0.001
Gender	0.915 (0.684-1.291)	0.612		
Smoke	1,147 (0.792-1.660)	0.468		
sCAD	1.897 (1.337-2.691)	<0.001		
Diabetes	1.630 (1.123-2.366)	0.010	1.588 (1.077-2.343)	0.020
Total cholesterol	1.003 (0.842-1.193)	0.976		
Triglycerides	1.258 (1.056-1.499)	0.010		
24 h-SBP	1.011 (0.999-1.023)	0.076		
24 h-DBP	0.994 (0.977-1.010)	0.442		

95% CI, 95% confidence interval; DBP, diastolic blood pressure; OR, odds ratio; sCAD, stable coronary artery disease; SBP, systolic blood pressure.
